# Scale Effects on the Ballistic Penetration of Graphene Sheets

**DOI:** 10.1038/s41598-018-25050-2

**Published:** 2018-04-30

**Authors:** Rafael A. Bizao, Leonardo D. Machado, Jose M. de Sousa, Nicola M. Pugno, Douglas S. Galvao

**Affiliations:** 1 Instituto de Física Gleb Wataghin, Universidade Estadual de Campinas, 13083-970 Campinas, SP Brazil; 2 Department of Civil, Environmental and Mechanical Engineering, Laboratory of Bio-Inspired and Graphene Nanomechanics, University of Trento, via Mesiano, 77, 38123 Trento, Italy; 3 Departamento de Física Teórica e Experimental, Universidade Federal do Rio Grande do Norte, Natal-RN, 59072-970 Brazil; 4 Departamento de Física, Universidade Federal do Piauí, Teresina, Piauí 64049-550 Brazil; 5 Ket-Lab, Edoardo Amaldi Foundation, Italian Space Agency, Via del Politecnico snc, 00133 Rome, Italy; 6 School of Engineering and Materials Science, Queen Mary University of London, Mile End Road, London, E1 4NS United Kingdom

## Abstract

Carbon nanostructures are promising ballistic protection materials, due to their low density and excellent mechanical properties. Recent experimental and computational investigations on the behavior of graphene under impact conditions revealed exceptional energy absorption properties as well. However, the reported numerical and experimental values differ by an order of magnitude. In this work, we combined numerical and analytical modeling to address this issue. In the numerical part, we employed reactive molecular dynamics to carry out ballistic tests on single, double, and triple-layered graphene sheets. We used velocity values within the range tested in experiments. Our numerical and the experimental results were used to determine parameters for a scaling law. We find that the specific penetration energy decreases as the number of layers (N) increases, from ∼15 MJ/kg for N = 1 to ∼0.9 MJ/kg for N = 350, for an impact velocity of 900 m/s. These values are in good agreement with simulations and experiments, within the entire range of N values for which data is presently available. Scale effects explain the apparent discrepancy between simulations and experiments.

## Introduction

The combination of very high Young’s modulus (1 TPa), ultimate strength (130 GPa), and low density values (≈2200 kg.m^−3^) makes graphene an ideal candidate material for ballistic protection applications^1^. However, the rapid strain increase found in these applications can lead to unexpected behavior. For instance, experiments in this regime revealed unzipping of carbon nanotubes (CNTs) into nanoribbons^2^. While the high-strain-rate behavior of CNTs, either isolated^3,4^ or in composites^5–8^, has been studied for years, investigations on graphene mainly date from 2014^9–16^. Of particular interest is the study by Lee *et al*.^9^, in which silica spheres were shot at multilayered graphene sheets. Exceptional energy absorption capabilities were found: the specific penetration energy of graphene was ten times greater than that of macroscopic steel. This was due in part to the impact energy being dissipated over an area much larger than that of the projectile cross-section.

Follow-up molecular dynamics (MD) studies elucidated the atomistic structures formed during penetration of graphene monolayers and the role played by defects^13^, determined the propagation velocity of the impact-induced stress wave^14^, and studied the failure mechanism of the graphene sheets^15^. These simulations also revealed extremely high specific energy penetration values, an order of magnitude greater than those measured in experiments. Up to now this large discrepancy between theory and experiment has remained unexplained. In this work, we combined fully atomistic reactive MD simulations and analytical modeling to address this issue.

## Results and Discussions

### Simulated ballistic tests

In the MD part of our study, we shot metallic projectiles at single, bilayer, and trilayer graphene sheets. We have considered different projectile velocities and impact angles, as well as sheets and projectiles of different dimensions (up to 400,000 atoms). As further discussed below, we also obtained MD specific penetration energy values that are one order of magnitude larger than those from experiments, but the difference decreased progressively for the cases with two and three layers. From these results, we were able to extract parameters to apply in a scaling law proposed by Pugno^17^. Our analytical model fits well all existing results for graphene.

**Figure 1:**
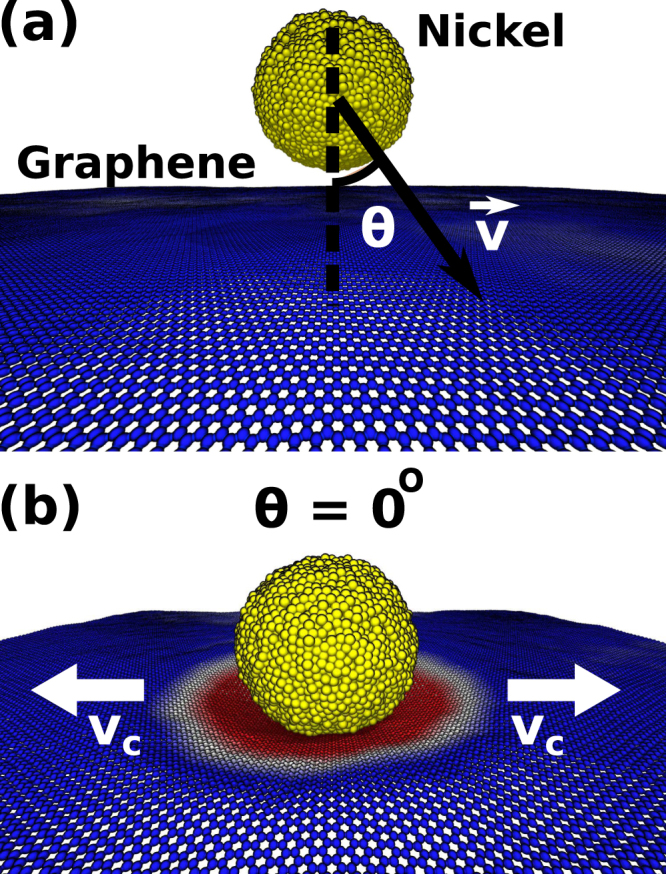
(**a**) Setup employed in the fully atomistic molecular dynamics (MD) simulations. We shot a nickel particle against graphene sheets, at different velocity **v** and angle *θ* values. (**b**) MD snapshot from a case with *θ* = 0° and *v* = 900 m/s. The ballistic impact generates an elastic deformation wave that propagates with velocity *v*_*c*_ over an area much larger than the particle dimensions.

A typical setup used in our ballistic tests is presented in Fig. 1a. The considered graphene targets were periodic along the planar directions, and ranged from 20 nm × 20 nm (30,000 atoms) to 100 nm × 100 nm (385,000 atoms). We have also considered structures with two and three layers. For these tests, we employed 40 nm × 40 nm graphene sheets and spherical nickel projectiles with a diameter (*d*) of 140 Å. For other simulations, a spherical ($d\sim 70$ Å) Ni nanoparticle was used as projectile. Different **v** and ***θ*** values were considered (see Fig. 1a). We also considered impacts with varying azimuthal angles. Detailed information regarding the simulations can be found in the Methods section.

**Figure 2:**
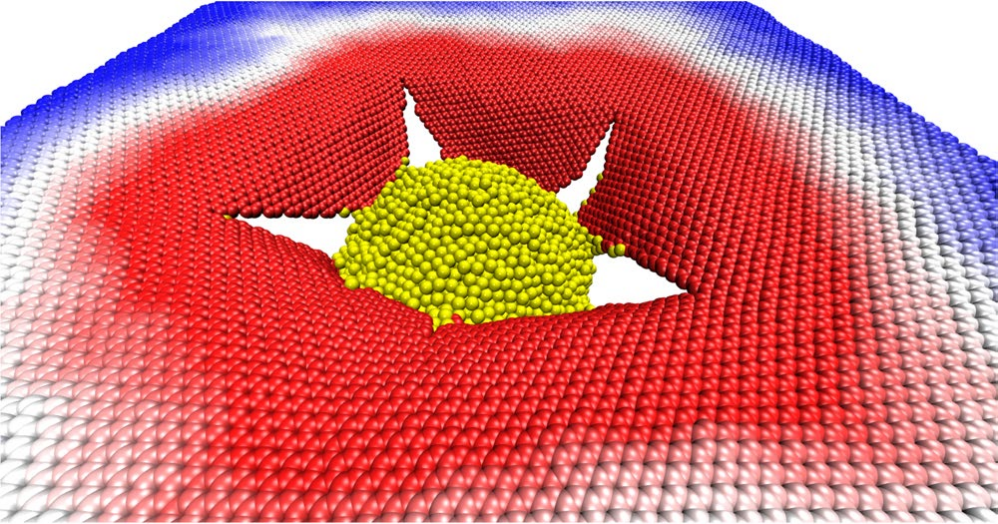
MD snapshot from a case of *θ* = 0° and *v* = 900 m/s showing the fractured graphene sheet after the ballistic impact.

**Figure 3:**
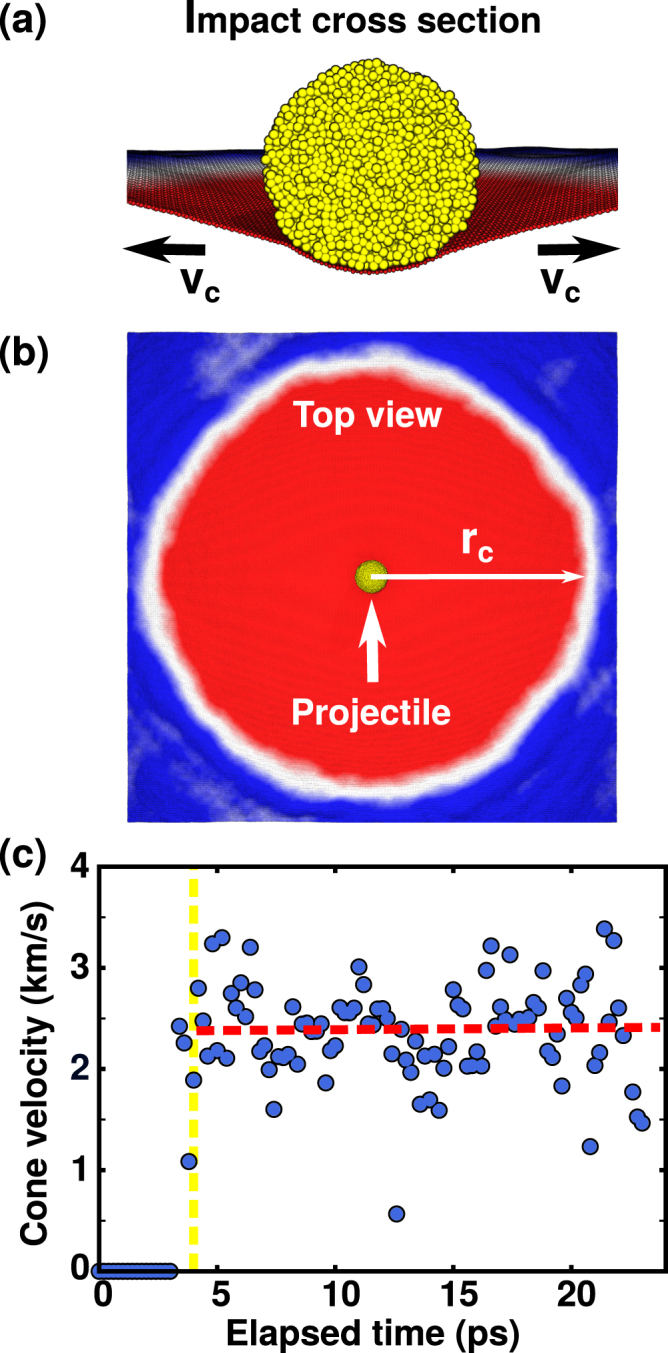
Results obtained for *θ* = 0° and *v* = 900 m/s. (**a**) Impact cross sectional view, showing a graphene sheet deformed into a conical shape. (**b**) Top view of an impact. Observe that the deformation cone radius (*r*_*c*_) is far larger than the projectile cross-section value. (**c**) Instantaneous cone velocity values. The linear fit (red line) suggests that, considering error bar fluctuations, the generated conical shape propagates at constant velocity. The points considered in the fit are to the right of the yellow line (impact time).

In Figs 1b and 2 we present MD snapshots for the case with *θ* = 0° and *v* = 900 m/s. In Figs 1, 2 and 3, graphene atoms are colored according to their z (height) coordinate values: positive values are in blue and negative ones in red. After impact, the generated elastic deformation wave propagates radially outwards with velocity *v*_*c*_ - see Fig. 1b. In agreement with the report by Lee *et al*.^9^, we observed deformation areas far larger than the projectile cross-section (Fig. 3b). Our typical fracture patterns are also consistent with experimental results^9^. For a better visualization of the whole process see videos in the Supplementary Information.

From our MD trajectories we can analyze in detail the onset and propagation of the impact-generated elastic deformation wave. Inspection of the cross-sectional view of an impact event (Fig. 3a) reveals that graphene stretches to accommodate the incoming projectile into a cone shape. Lee *et al*.^9^ reached the same conclusion from their experiments and estimated, using the formula proposed by Phoenix and Porwal^18^, a velocity of *v*_*c*_ = 2560 m/s for an impact velocity of 900 m/s. From our MD trajectories we can not only calculate average cone velocities, but also their time evolution. In our analysis, atoms that moved 12 Å down from their initial position were assumed inside the cone. The first atoms to cross this threshold were considered at the impact center, and for every MD snapshot frame we calculated the distance from this center to the farthest atom in the cone, *r*_*c*_ (see Fig. 3b). If the time between adjacent frames is Δ*t* and the cone radius increased by Δ*r*_*c*_ in this interval, the instantaneous velocity can be calculated by using *v*_*c*_ = Δ*r*_*c*_/Δ*t*.

Results of this analysis are presented in Fig. 3c, where the red dotted line is a linear fit of the data. More details are discussed in the Supplementary Information. For the case presented in Fig. 3c, we obtained a cone acceleration of 0.0017 ± 0.0095 km/s^2^. Near zero acceleration values were also observed for other impact velocities, indicating that the cones propagated with constant velocity for all the analyzed events. For an impact at 900 m/s, we found *v*_*c*_ = 2.37 ± 0.14 km/s, a value rather close to the estimation by Lee *et al*.^9^. Graphs for other impact velocities are also presented in the Supplementary Information. For an impact at 600 (1100) m/s we obtained average cone velocities of 1.99 ± 0.15 (2.64 ± 0.10) km/s, values that are again close to those estimated by Lee *et al*., 1.95 (2.92) km/s^9^. It should be remarked that Haque *et al*.^14^ also found constant *v*_*c*_ values in their MD simulations. However, under the higher velocity conditions they used, the obtained *v*_*c*_ values were $\sim \mathrm{35 \% }$ lower than those obtained by employing the formula by Phoenix and Porwal^18^, suggesting a limit of validity for this expression.

In order to contrast our results against other theoretical^13–15^ and experimental^9^ reports, we normalized the absorbed energy by the graphene mass within the projectile cross-sectional area, obtaining the Specific Penetration Energy (SPE). This comparison is presented in Table 1. In the Methods section, we discuss details of the approach we used to determine: (i) the energy absorbed by graphene during impact, and (ii) the SPE values attributed to Haque *et al*.^14^. Note that currently reported numerical values are an order of magnitude larger than experimental amounts, although the difference decreased for the considered bilayer and trilayer systems. It is important to remark that direct comparison between numerical (up to now single, bilayer, and trilayer systems) and experimental (up to now from 30 up to 300 layers) results is not presently possible, due to computational/technological limitations.

**Table 1:** Specific penetration energy values.

Velocity (m/s)	Number of layers	Specific penetration energy (MJ/kg)
900	1	15.0 (MD)
2000	1	23.6^15^ (MD)
5000	1	29.0^13^ (MD)
5000	1	40.8^14^ (MD)
900	2	13.4 (MD)
5000	2	25.2^14^ (MD)
900	3	10.2 (MD)
600	127 (average)	1.09^9^ (EXP)
900	154 (average)	1.26^9^ (EXP)

We have also carried out tests to investigate whether our results could be affected by the limited size of the investigated structures. To this end, we examined the collision of a Nickel projectile (*d* = 70 Å, *θ* = 0°, and *v* = 900 m/s) against square graphene sheets of varying length (20 and 40 nm). We observed reflection of impact-generated elastic waves at the system boundaries in both cases. For the larger structure, however, waves returned to the impact region only after fracture completion. See videos in the Supplementary Information. The smaller structure also absorbed less energy during these ballistic tests: increasing system size increased SPE from 12.9 MJ/kg to 14.1 MJ/kg. Regarding the structures employed to obtain the SPE values presented in Table 1, we also observed waves returning to the impact region while fracturing was underway. This result is not surprising, as we employed larger sheets but also larger projectiles in those simulations. This analysis indicates that the provided SPE values are likely underestimated, but increased SPE values would not modify the main conclusions of the present work. The computational cost of performing ballistic tests on even larger graphene sheets is currently prohibitive.

### Scaling law

The lowered specific penetration energy in tests with two or three layers suggests a dependency of this quantity with the number of layers, and that a size-effect rescaling is needed in order to contrast numerical and experimental results. Note this effect can also be observed in the results provided by Haque *et al*.^14^. In order to investigate this possibility, we applied the scaling law proposed by Pugno^17^ to correlate results across different scales. The key to understanding these results is that the strength of a material subject to nanoindentation or tensile tests has been, under fairly general assumptions, shown to be a function of its structural size^17^.

The model we used was originally derived for ductile materials, considering that a certain number of dislocations is necessary to generate plastic deformation in a material, with a particularity that the density of dislocations was limited at nanoscale to avoid divergences observed in past models^17,19,20^. This limit is based on the fact that materials must have finite strength, and improves the connection between results obtained at different system sizes. Thereafter, the model was extended to fit brittle materials as well, and its validity at different size scales was observed^17^.

Considering a spherical projectile, we can write down the strength *σ*_*N*_ of an *N*−layered material as a function of its strength at the macroscale^17^

1 $${{\rm{\sigma }}}_{N}={{\rm{\sigma }}}_{\infty }\sqrt{1+\frac{{N}_{c}}{N+{N}_{c}^{^{\prime} }}},$$

where σ_∞_ is the macroscale strength of the bulk material, while *N*_*c*_ and ${N}_{c}^{^{\prime} }$ are characteristic numbers to be determined and describing the nanoscale strength and the transition from the nano- to the macro-scale. In our work, all three quantities were obtained from numerical and experimental ballistic results. More details on the model derivation can be found in ref.^17^.

One possible way to define the specific penetration energy of an *N*-layered material is

2 $${d}_{N}=\frac{E}{\rho {A}_{p}Nt},$$

in which *A*_*p*_, *ρ*, *N*, *t* are respectively the projectile cross section area, density, number of layers and thickness of the single layer. This quantity can be related to the specific strength of the *N*-layered material (σ_*N*_)^21,22^. See the Supplementary Information for more details on this procedure. Thus, we can write

3 $${d}_{N}=\frac{{{\rm{\sigma }}}_{N}}{\eta \rho },$$

in which *η* is the ratio between the area of the projectile cross-section and the area of the damaged zone. This number is lower than one if an area larger than the cross-sectional area of the projectile is uniformly impacted. Notice that we are determining an expression for fixed impact velocity, as *d*_*N*_ should also depend on *v*^9^. An issue from our MD simulations is that the projectile changes velocity during impact. This change is observed in experiments as well: in one instance, the measured projectile kinetic energy decreased from 9 nJ to 4.5 nJ^9^. In order to mitigate this issue, we considered large projectiles with *d* = 140 Å.

For instance, by using graphene density (*ρ* ≈ 2200 kg. m^−3^) and our *d*_1_ and *d*_2_ simulation values for *v* = 900 m/s, we can derive σ_1_ and σ_2_ from Eq. 3

4 $${{\rm{\sigma }}}_{1}={d}_{1}\eta \rho =33.0\,{\rm{GPa}}$$

5 $${{\rm{\sigma }}}_{2}={d}_{2}\eta \rho =29.5\,{\rm{GPa}},$$

where we considered *η* = 1.

A general expression for the specific penetration energy can be found from equations 1 and 3,

6 $${d}_{N}={d}_{\infty }\sqrt{1+\frac{{N}_{c}}{N+{N^{\prime} }_{c}}},$$

where *d*_∞_ = *σ*_∞_/*ηρ*.

**Figure 4:**
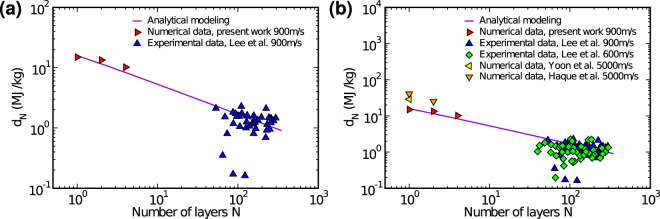
(**a**) Analytical modeling fitting numerical results from molecular dynamics simulations carried out at the nanoscale and experimental ballistic test results carried out at the microscale by Lee *et al*.^9^, for an impact velocity of *v* = 900 m/s. (**b**) Comparison between our analytical model and results for other impact velocities. Notice data points obtained at higher/lower velocities are located above/below the analytical modeling curve. In spite of that, the overall trend is a decrease in *d*_*N*_ as more layers are considered.

We can fit previous^9^ and current results with equation 6 to estimate the parameters *d*_∞_, *N*_*c*_ and ${N}_{c}^{^{\prime} }$. Running a 100 iterations best fit with tolerance 10^−5^ we found *d*_∞_ = 0.05 MJ/kg, *N*_*c*_ = 134737 and ${N}_{c}^{^{\prime} }=0.14$. After all parameters are obtained, we can use equation Eq. 6 to estimate the specific penetration energy for any number of layers. The values obtained for few-layer graphene sheets are an order of magnitude higher than those obtained in the microscale, suggesting a very sharp transition in the scaling law - see Fig. 4. Other simulation results^13,14^ are also presented for comparison. Since the highest energy absorption per affected graphene mass is obtained when *N* is small, thin graphene nanocoatings could be employed to maximize this quantity in ballistic applications. Note that solid substrates could affect the performance of nanocoatings, by preventing out-of-plane deformation of the graphene sheets. This could be avoided by using low-density substrates, such as graphene sponges, which can present densities similar to air^23^. Graphene coated sponges have already been applied in oil absorption^24^.

**Figure 5:**
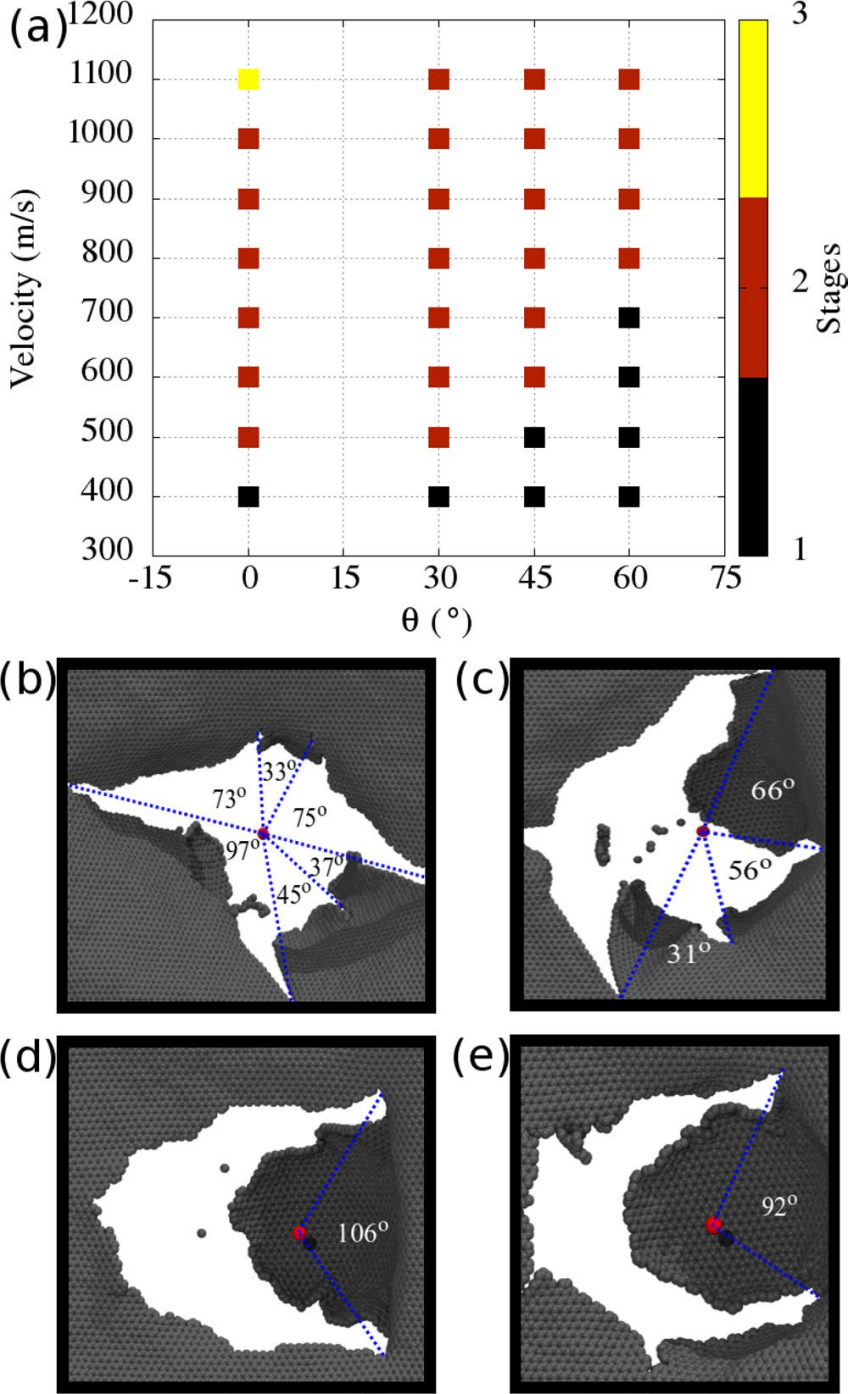
(**a**) Summary of the results for varied impact angles and velocities. There are three basic outcomes: (1/black) the projectile bounces back, without damage to the graphene sheet; (2/red) the projectile fractures the graphene sheet, but is unable to overcome the mutual van der Waals attraction, eventually coming to a full stop; (3/yellow) the projectile pierces through the graphene sheet. (**b–d**) Fracture patterns after impact for angle values of (b) *θ* = 0°, (c) *θ* = 30°, (d) *θ* = 45°, and (e) *θ* = 60°. Also indicated in these snapshots are the angle values between adjacent cracks.

We also considered non perpendicular projectile impacts against single layer graphene sheets. Summary of the results for collisions with *θ* ≠ 0° are presented in Fig. 5a. We observed that collisions are rather elastic for higher impact angles and lower velocities; the projectile may even bounce back. Fracture occurs for outcomes (2) and (3). Inspection of Fig. 5a reveals that for higher impact velocities fracture occurs regardless of the impact angle. Fracture patterns for different *θ* values are presented in Fig. 5b–e. As previously mentioned, our patterns for *θ* = 0° are in good agreement with those reported by Lee *et al*.^9^, regarding both petal quantity and average opening angle between them. This suggests fracture patterns are scale independent, increasing the reliability of the predicted patterns presented for alternate impact angles in Fig. 5c–e.

## Conclusions

In summary, we combined MD simulations and analytical modeling to explain the apparent discrepancies between numerical and experimental results for the specific penetration energy of graphene under ballistic impact. In the MD part of this work, we shot nickel projectiles at varied angles and velocities against single, double, and trilayer graphene sheets, and studied the resulting dynamics and fracture patterns. Our results for perpendicular impacts were in good agreement with experimental data, suggesting these patterns are scale independent. The values we obtained for specific penetration energy from these simulations were consistent with previous numerical reports for single-layer graphene^13^, but were an order of magnitude greater than experimental values for multi-layer sheets^9^. Our analytical model suggests this disparity is due to size-scale effects, and the proposed power law was able to produce an excellent fitting of the numerical and experimental results obtained in different scale regimes. Our results also suggest that superior performance per graphene mass can be obtained in ballistic applications by applying thin nanocoatings over other materials.

## Methods

### Computational Methods and details

Our molecular dynamics (MD) simulations were carried out using the Reactive Force Field (ReaxFF)^25,26^, as implemented in the LAMMPS software package^27^. We used the parametrization described in Mueller *et al*.^28^. ReaxFF is a reactive force field parametrized using ab-initio methods. It allows for the formation and dissociation of chemical bonds, making it potentially applicable to simulation of fractures at the nanoscale.

As the projectile, we used nickel nanoparticles of varying size. For the results presented in Fig. 4 and Table 1, we employed a 112000 atoms spherical nanoparticle with a diameter of 140 Å. For the other ballistic tests, we employed a 14000 atom nanoparticle, packed into a $\sim 70$ Å diameter sphere. As the target, we used periodic graphene sheets, ranging from 20 nm × 20 nm (30000 atoms) to 100 nm × 100 nm (385000 atoms). For tests carried out with the larger projectiles, we employed 40 nm × 40 nm graphene sheets with one, two, or three layers.

We employed the following procedure in our simulations:

1. We minimized and thermalized the nickel nanoparticle for 200 ps at 300 K in the *NVT* ensemble
2. We minimized and thermalized the graphene sheet for 200 ps at 300 K in the *NPT* ensemble. To reduce the initial stress, we set a null pressure at the edges of the structure
3. We thermalized the graphene sheet for an additional 200 ps at 300 K in the *NVT* ensemble
4. We fixed the edges of the graphene unit cell, to prevent uniform translation of the sheet during impact
5. We shot the projectile against the graphene sheet in the *NVE* ensemble, with velocity **v** and angle ***θ***. Different **v** and ***θ*** values were considered.

For steps 1 to 3 we used a timestep of 0.5 fs while in step 5 we used a timestep of 0.02 fs. Temperature and pressure were controlled through chains of three Nosé-Hoover thermostats and barostats^29^.

We would like to stress that the used simulation setup was devised to mimic (within computational limitations) the experimental conditions used to investigate the mechanical behavior of graphene under high strain-rate conditions. In the experiments, graphene is also suspended and its edges are glued to a sample holder.

For collisions considering an angle *θ* ≠ 0 or *ϕ* ≠ 0, in some instances the vertical deformation reached the fixed end. In those cases, boundary effects may have played a role in determining the final outcome. For ballistic tests with varied azimuthal angle (*ϕ*) of impact, we fixed the velocity (*v* = 1100 m/s) and the polar angle (*θ* = 30°) of impact. We considered *ϕ* values of 0°, 15°, 30°, 45°, and 60°.

In order to calculate the energy absorbed by graphene, we first determine the change in the projectile’s kinetic (Δ*E*_*kin*,*projectile*_) and potential (Δ*E*_*pot*,*projectile*_) energies. The energy absorbed by graphene is minus their sum^13^: Δ*E*_*graphene*_ = −Δ*E*_*projectile*_ = −(Δ*E*_*kin*,*projectile*_ + Δ*E*_*pot*,*projectile*_).

### Procedure to calculate the specific penetration energy from the data published by Haque *et al*

In that paper, the energy transferred to the graphene sheet during a ballistic test is ${E}_{T}^{GS}$. In order to obtain the specific penetration energy (*d*_*N*_), this energy has to be divided by the graphene mass within the projectile cross section. This mass is equal to *m* = *πR*^2^*N*_*L*_*ρ*_*A*_, where *R* is the projectile radius, *N*_*L*_ is the number of layers, and *ρ*_*A*_ = 0.77 mg/m^2^ is the area density of graphene. For *v* = 5000 m/s, we obtained from the manuscript that ${E}_{T}^{GS}=36.13$ aJ for *N*_*L*_ = 1 and ${E}_{T}^{GS}=44.65$ aJ for *N*_*L*_ = 2. After dividing these results by the mass, we get *d*_1_ = 40.8 MJ and *d*_2_ = 25.2 MJ. More ${E}_{T}^{GS}$ data is presented in the paper, but this is the only velocity for which results are presented in which complete penetration is observed for different number of layers^14^.

### Procedure to extract data from Lee *et al*., Yoon *et al*., and Xia *et al*

In order to extract data from Fig. 4c of Lee *et al*.^9^, Fig. 4a of Yoon *et al*.^13^, and Fig. 9a of Xia *et al*.^15^, we used the web app WebPlotDigitizer^30^.

## Electronic supplementary material

Video 1

Video 2

Video 3

Video 4

Supplementary Information
