# Supplementary material for: Scale Effects on the Ballistic Penetration of Graphene Sheets
Source: Sci Rep. 2018 Apr 30;8:6750. doi: 10.1038/s41598-018-25050-2 (PMC5928051; doi:10.1038/s41598-018-25050-2)
Supplement: Supplementary file 5 — Supplementary Information [file 41598_2018_25050_MOESM5_ESM.pdf]

Supplementary Information: Scale Effects on the Ballistic Penetration of Graphene Sheets

Rafael A. Bizao,[1,2] Leonardo D. Machado,[1,3] Jose M. de Sousa,[1,4] Nicola M. Pugno,[2,5,6,*] and Douglas S. Galvao[1,†]

[1]*Instituto de Física Gleb Wataghin, Universidade Estadual de Campinas, 13083-970, Campinas, SP, Brazil.*

[2]*Department of Civil, Environmental and Mechanical Engineering, Laboratory of Bio-Inspired and Graphene Nanomechanics, University of Trento, via Mesiano, 77, 38123 Trento, Italy.*

[3]*Departamento de Física Teórica e Experimental, Universidade Federal do Rio Grande do Norte, Natal-RN 59072-970, Brazil*

[4]*Departamento de Física, Universidade Federal do Piauí, Teresina, Piauí, 64049-550, Brazil.*

[5]*Ket-Lab, Edoardo Amaldi Foundation, Italian Space Agency, Via del Politecnico snc, 00133 Rome, Italy.*

[6]*School of Engineering and Materials Science, Queen Mary University of London, Mile End Road, London E1 4NS, United Kingdom.*

(Dated: March 8, 2018)

CONTENTS

Additional information: cone propagation velocity calculations 2

Derivation of the equations 2

The effect of pre-tension in graphene layers during ballistic penetration 5

Azimuth angle dependence 6

Linear scale version of Figure 4 7

References 8

ADDITIONAL INFORMATION: CONE PROPAGATION VELOCITY CALCULATIONS

We attribute fluctuations in instantaneous cone velocity values to thermal effects. In order to employ conditions as close as possible to the experiments, we set the initial temperature of the system at $T = 300$ K. Because of this, the z position of every atom fluctuates during MD simulations. In the criteria employed, an atom is considered inside the deformation cone when its position is lower than a threshold ($z < 12$ Å). If an atom is fluctuating down, its instantaneous position might be lower than the threshold, although its equilibrium position is not. The reverse might occur if it is fluctuating upwards. There is thus some uncertainty in our criteria. Note also that relative fluctuations decrease as impact velocities increase, which can be correlated to an increased ratio between kinetic and thermal energies (compare figures S1 and S2). Our finding that deformation cones propagate at constant velocities is corroborated by Haque *et al.* [1], that set initial temperatures to $T = 1$ K to avoid such effects.

DERIVATION OF THE EQUATIONS

According to Pugno [2, 3], considering a collision generating large-sized fragments, the absorbed energy (E) is proportional to the volume dislocated during the collision process

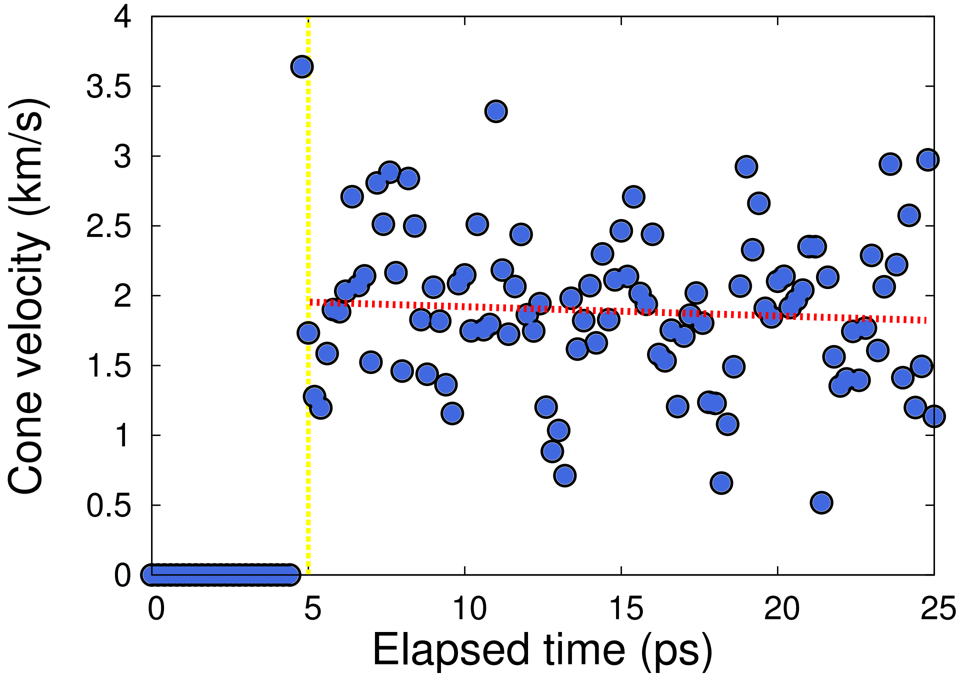

FIG. S1. Instantaneous cone velocity values, for $v = 600$ m/s and $\theta = 0°$. The linear fit (red line) suggests that, considering error bar fluctuations, the generated conical shape propagates at constant velocity. For this impact velocity, the cone acceleration is $a = -0.0066 \pm 0.0092$ km/s^2 and the cone velocity is $v = 1.99 \pm 0.15$ km/s. The points considered in the fit are to the right of the yellow line (impact time).

(V) and the proportionality constant is close to the strength of the material (σ), so we have

$$E = \sigma V. \tag{1}$$

From this equation it is possible to derive the strength of an N-layered material as

$$\sigma_N = \frac{E}{V} = \frac{E}{m}\rho, \tag{2}$$

where ρ is the density of the target material and m is the mass of the affected region, which in our case can be calculated as

$$m = \rho A_f N t, \tag{3}$$

where A_f, N and t are respectively the damaged zone area, number of layers and thickness of the single layer. For the thickness we used the well-known graphene thickness $t = 0.34$ nm. So we have

$$\sigma_N = \frac{E}{\rho A_f N t}\rho. \tag{4}$$

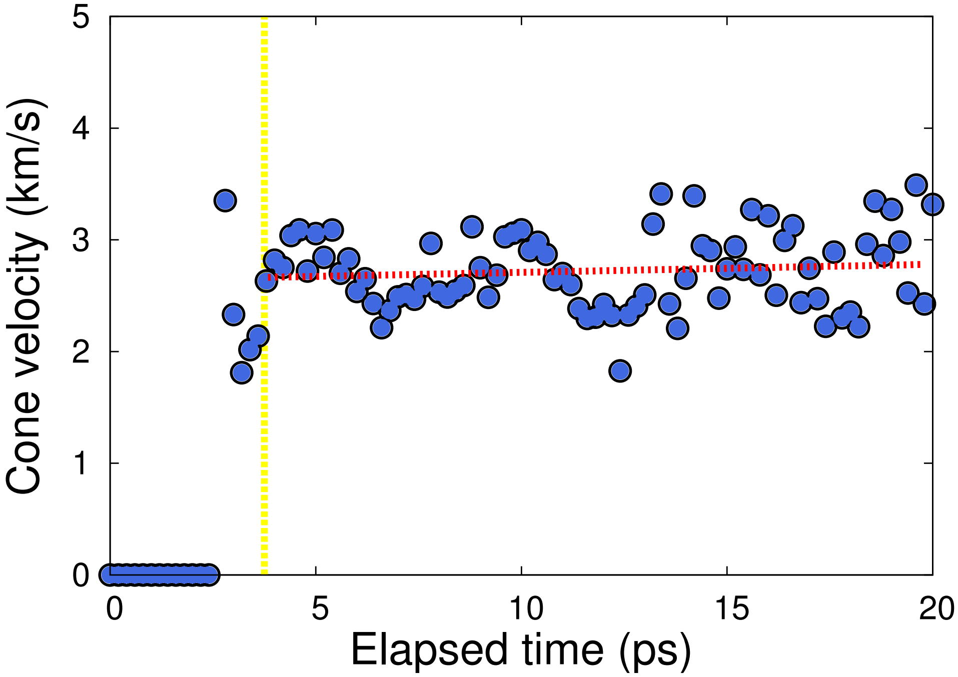

FIG. S2. Instantaneous cone velocity values, for $v = 1100$ m/s and $\theta = 0°$. The linear fit (red line) suggests that, considering error bar fluctuations, the generated conical shape propagates at constant velocity. For this impact velocity, the cone acceleration is $a = 0.0072 \pm 0.0079$ km/s^2 and the cone velocity is $v = 2.64 \pm 0.10$ km/s. The points considered in the fit are to the right of the yellow line (impact time).

Introducing the η parameter

$$\eta = \frac{A_p}{A_f}, \tag{5}$$

where $A_p = \pi r^2$ is the particle cross section area with r being the particle radius. So we end up with the equation presented in the paper

$$\sigma_N = d_N \rho \eta \tag{6}$$

where d_N

$$d_N = \frac{E}{\rho A_p N t} \tag{7}$$

is the well known specific penetration energy. Equation 7 allow us to relate the absorbed energy during collision with the strength of the target material, and to directly compare our d_N results with the literature.

To do the scale analysis we followed the procedure presented in Pugno [4] relating the strength of the material with its structural size, here number of layers N

$$\sigma_N = \sigma_\infty \sqrt{1 + \frac{N_c}{N + N_c^{'}}}, \tag{8}$$

where σ_∞ is the strength of the bulk material, while N_c and $N_c^{'}$ are critical values to be determined.

So combining equations 6 and 8 we obtain the equation presented in the paper

$$d_N = d_\infty \sqrt{1 + \frac{N_c}{N + N_c^{'}}}, \tag{9}$$

where $d_\infty = \sigma_\infty / \eta\rho$. From this equation it is possible to do a scale analysis considering the specific penetration energy instead of the strength of the material.

THE EFFECT OF PRE-TENSION IN GRAPHENE LAYERS DURING BALLISTIC PENETRATION

In this manuscript, we suggested that depositing graphene nanocoatings on low-density substrates (such as graphene sponges) would maximize specific penetration energy values. It should be noted, however, that in such a system graphene should not be pre-tensioned at the onset of impact. If that is the case, the estimated values of specific penetration energy could differ from those predicted here. Xia *et al.* showed that the shape of the graphene membrane used in a ballistic penetration test influences stress distribution, which in turn influences the specific penetration energy (SPE) [5]. As the absence of pre-tension is very likely to lead to different stress distribution at impact, it might also lead to modified values of specific penetration energy. Note, however, that the variations found by Xia et al. never exceeded 20%. We therefore expect our main conclusions to remain valid.

AZIMUTH ANGLE DEPENDENCE

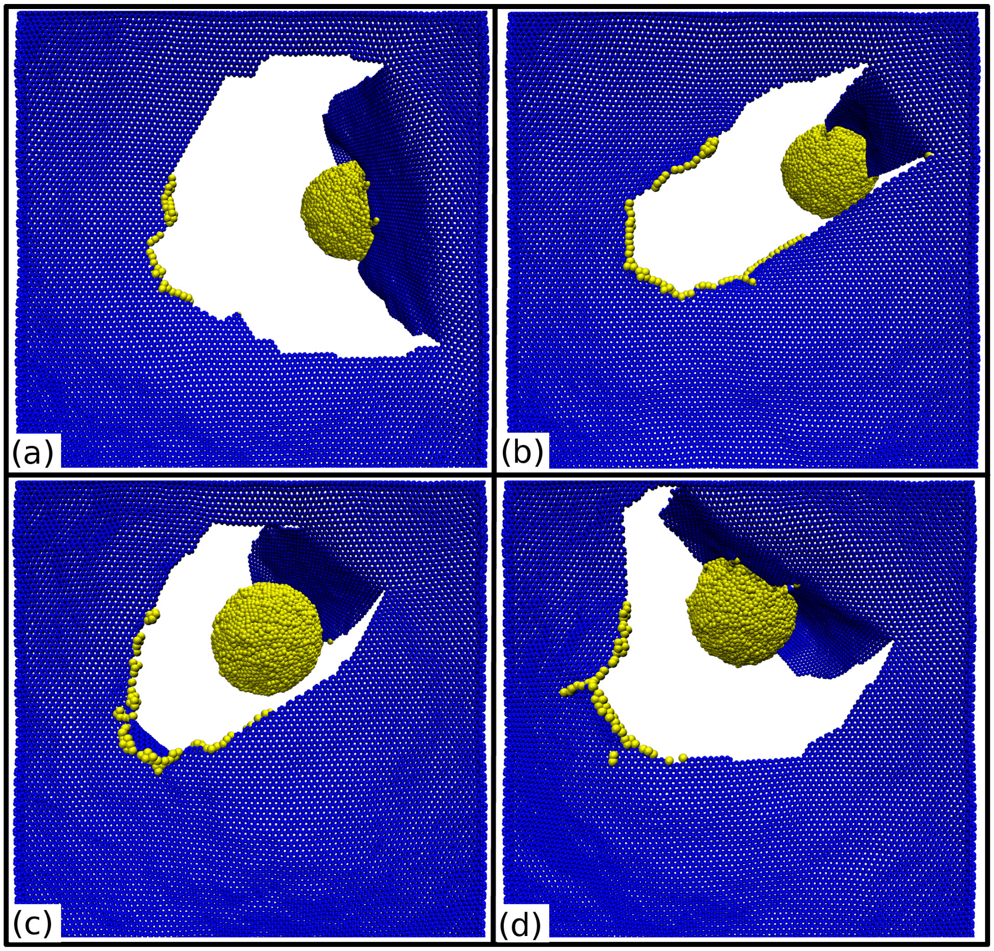

FIG. S3. Results of ballistic tests where we fixed the impact velocity ($v = 1100$ m/s), the polar angle ($\theta = 30°$), and varied the azimuth angle (ϕ). The tested ϕ values were (a) 15°, (b) 30°, (c) 45°, and (d) 60°. Note that fracture patterns were more localized for $\phi = 30°$ and $\phi = 45°$.

LINEAR SCALE VERSION OF FIGURE 4

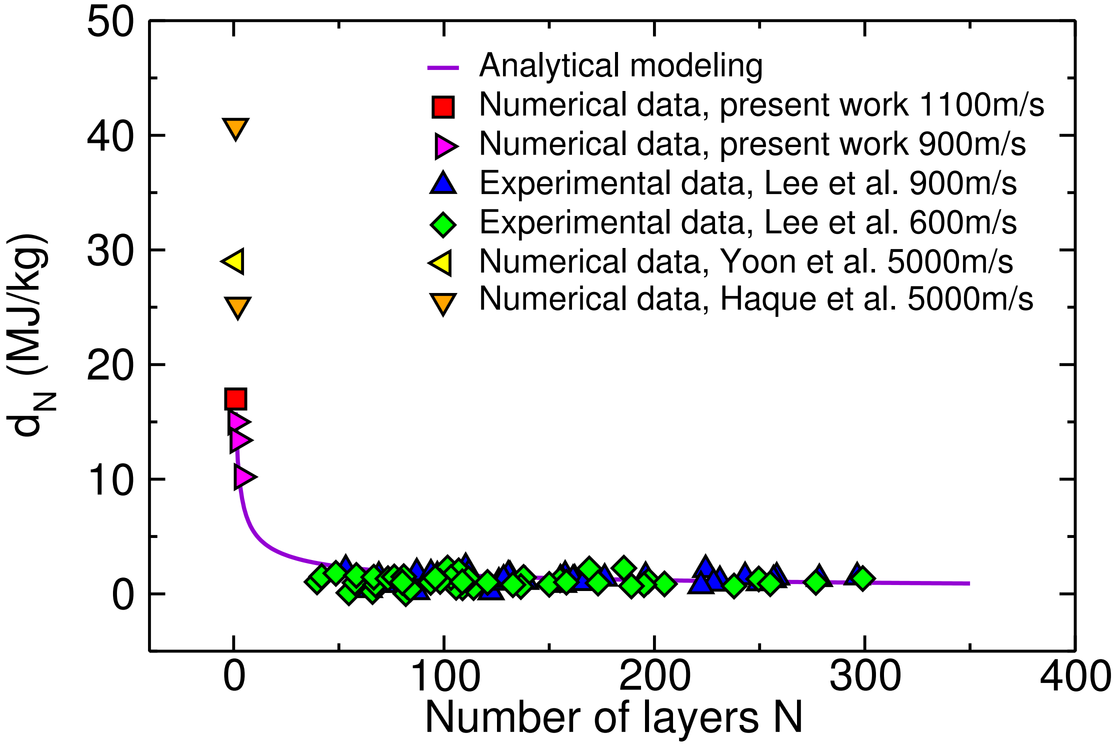

FIG. S4. Alternative version of Figure 4, in which the same results are presented in a linear plot.

* nicola.pugno@unitn.it

† galvao@ifi.unicamp.br

[1] B. Z. G. Haque, S. C. Chowdhury, and J. W. Gillespie, Carbon **102**, 126 (2016).

[2] N. M. Pugno, Journal of applied mechanics **73**, 697 (2006).

[3] A. Carpinteri and N. Pugno, Journal of Applied Mechanics **69**, 854 (2002).

[4] N. M. Pugno, Acta Materialia **55**, 1947 (2007).

[5] K. Xia, H. Zhan, D. Hu, and Y. Gu, Scientific Reports **6**, 33139 (2016).
